# The transcriptome analysis on urea response mechanism in the process of ergosterol synthesis by *Cordyceps cicadae*

**DOI:** 10.1038/s41598-021-90377-2

**Published:** 2021-05-25

**Authors:** Qihui Su, Zhicai Zhang, Xiaocui Liu, Feng Wang

**Affiliations:** 1 School of Food Science and Biological Engineering, Jiangsu University, Zhenjiang, 212013 People’s Republic of China; 2 Institute of Agro-Production Processing Engineering, Jiangsu University, Zhenjiang, 212013 People’s Republic of China; 3 Zhenjiang Yemaikang Food Bio-Technology Co., Ltd, Zhenjiang, 212013 People’s Republic of China

**Keywords:** Fungal genomics, Metabolomics

## Abstract

Nitrogen source is required for the growth of *Cordyceps cicadae* and involved in the regulation of metabolite synthesis. In order to further investigate the regulatory effects of nitrogen sources on the ergosterol synthesis by *C. cicadae*. We first confirmed that urea could significantly increase the ergosterol synthesis. The transcriptome analysis showed that compared with biomass cultured in the control fermentation medium (CFM), 1340 differentially expressed genes (DEGs) were obtained by Gene Ontology (GO) annotation, and 312 DEGs were obtained by Kyoto Encyclopedia of Genes and Genomes (KEGG) annotation from the biomass cultured in CFM + CO(NH_2_)_2_. Urea up-regulated D-3-phosphoglycerate dehydrogenase gene transcription level and down-regulated enolase and L-serine/L-threonine ammonialyase gene transcription level, increased serine synthesis, allosterically activate pyruvate kinase, to promote the synthesis of pyruvate and CH_3_CO ~ SCOA, the primer of ergosterol; Urea increase the genes transcription related with ergosterol synthesis by up-regulating the steroid regulatory element binding protein gene transcription levels. The transcriptome results were provided by those of qRT-PCR. Collectively, our finding provided valuable insights into the regulatory effect of nitrogen source on the ergosterol synthesis by *C. cicadae*.

## Introduction

As a parasitic fungus that can grow on the nymph of *Cicada flammata* Distant, *Platypleura kaempferi* Fabricius, *Crytotympana pustulata* Fabricous^1^, *Cordyceps cicadae* Shing (Chanhua) belongs to genus *Cordyceps* (family Clavicipitaceae, Ascomycotina). *C. cicadae* is a source of a rare traditional Chinese medicine that has been applied to treat childhood palpitation, epilepsy, convulsions, and several types of eye diseases^2^. *C. cicadae* is rich in sphingolipids^3^, polysaccharides^4^, nucleosides^5^, mannitol^6^, ergosterol^7^ and other active substances. Modern medical research has demonstrated that *C. cicadae* possesses more potent immunoregulatory^8^ and renal functions^9^ as well as anti-diabetic^10^, anti-bacterial^11^ and anti-tumorigenic properties^12^. Ergosterol, one of chemical components from mycelium cells, is the predominant sterol found in most fungi^13^. It can be divided into free ergosterol and esterified ergosterol^14^. Ergosterol mainly involves in the composition of microbial cell membranes, ensuring cell membrane integrity. In recent years, ergosterol has attracted an increasing interest because of its well-known anti-inflammatory^15^, anti-tyrosinase^16^ and anti-cancer^17^ activities, together with their applications in new drug formulations with antibiotics^18^. Yajaira et al. have found that ergosterol exerts synergistic effects on cell proliferation^19^. Slominski et al. have confirmed that ergosterol can be metabolized in vivo to generate new bioactive products, which have been found to be able to inhibit the proliferation of skin cells in culture^20^.

Due to limit of nature sources, the liquid fermentation has become a major approach to obtain active biomedical fungi. In the liquid fermentation process, the nitrogen source is one of main nutrient components and involves in synthesis of protein, nucleic acid, vitamin and so on. However, the effects of different nitrogen sources on the yield of the metabolite in the fermentation process are different. After different amino acid, nucleotide, (NH_4_)_2_SO_4_ and CO(NH_2_)_2_ are used as nitrogen sources, Su et al. have found that the nitrogen source plays a more relevant role in controlling the growth of the *S. cerevisiae* and *T. delbrueckii* yeasts, and the optimum nitrogen sources possess a relatively shorter lag phase time, a higher maximum growth rate and a higher maximum dissolved oxygen^21^. *Klebsiella* sp. SQY5 degrades tetracycline, and the removal rates of tetracycline are 1.97 mg/L and 0.97 mg/L, respectively, when NO_3_^-^ and NH_4_^+^ are used as nitrogen sources^22^. Furthermore, Shao et al. have found that specific functional genes participate in the tetracycline degradation, including energy production and conversion, amino acid transport and metabolism, when NO_3_^-^ and NH_4_^+^ are used as the nitrogen sources^22^. In the erythromycin fermentation of *Saccharopolyspora erythraea*, the excessive initial concentration of ammonium sulfate in the medium reduces the yield of erythromycin. However, a high concentration of ammonium nitrate can increase the yield of erythromycin^23^. To the best of our knowledge, only few studies have focused on the regulatory effects of nitrogen sources on medicinal fungi.

Steroid regulatory element binding protein (SREBP) is a family of membrane-bound transcription factors belonging to basic helix-loop-helix (BHLH) family. Since the discovery of SREBP in mammalian cells, SREBP or SREBP-like genes have also been found in plants and fungi. After analyzed 530 fungi, Ruan et al. found 367 fungi had SREBP, and identified 641 SREBPs^24^. Many fungi had more than two SREBP genes. Under normal conditions, SREBP is anchored to the endoplasmic reticulum membrane by SREBP cleavage-activating protein (SCAP) , but SREBP is activated by multiple proteins when ergosterol content is decreased or oxygen is deficient in vivo, SREBP is rapidly transferred from the endoplasmic reticulum to the Golgi apparatus with the help of SCAP, and the n-terminal of SREBP is released and enters into the cell nucleus and bounds to sterol regulatory element (Sre) to produce positive feedback activation, enhanced transcription^24–26^. It is unknown whether SREBP exists in the intracellular of *C.cicadae* and which factors can activate SREBP.

Few studies have paid attention to the adjustment effects of nitrogen sources on the metabolite synthesis. In the present study, we investigated the effects of different nitrogen sources on the intracellular and extracellular ergosterol levels. Furthermore, genome difference in the presence of CO(NH_2_)_2_ was analyzed using the medium in the absence of nitrogen sources as the control. Collectively, our findings greatly contributed to the increase of ergosterol yield in the fermentation process of *C. cicadae*.

## Results and discussion

### Effects of various nitrogen sources

**Figure 1:**
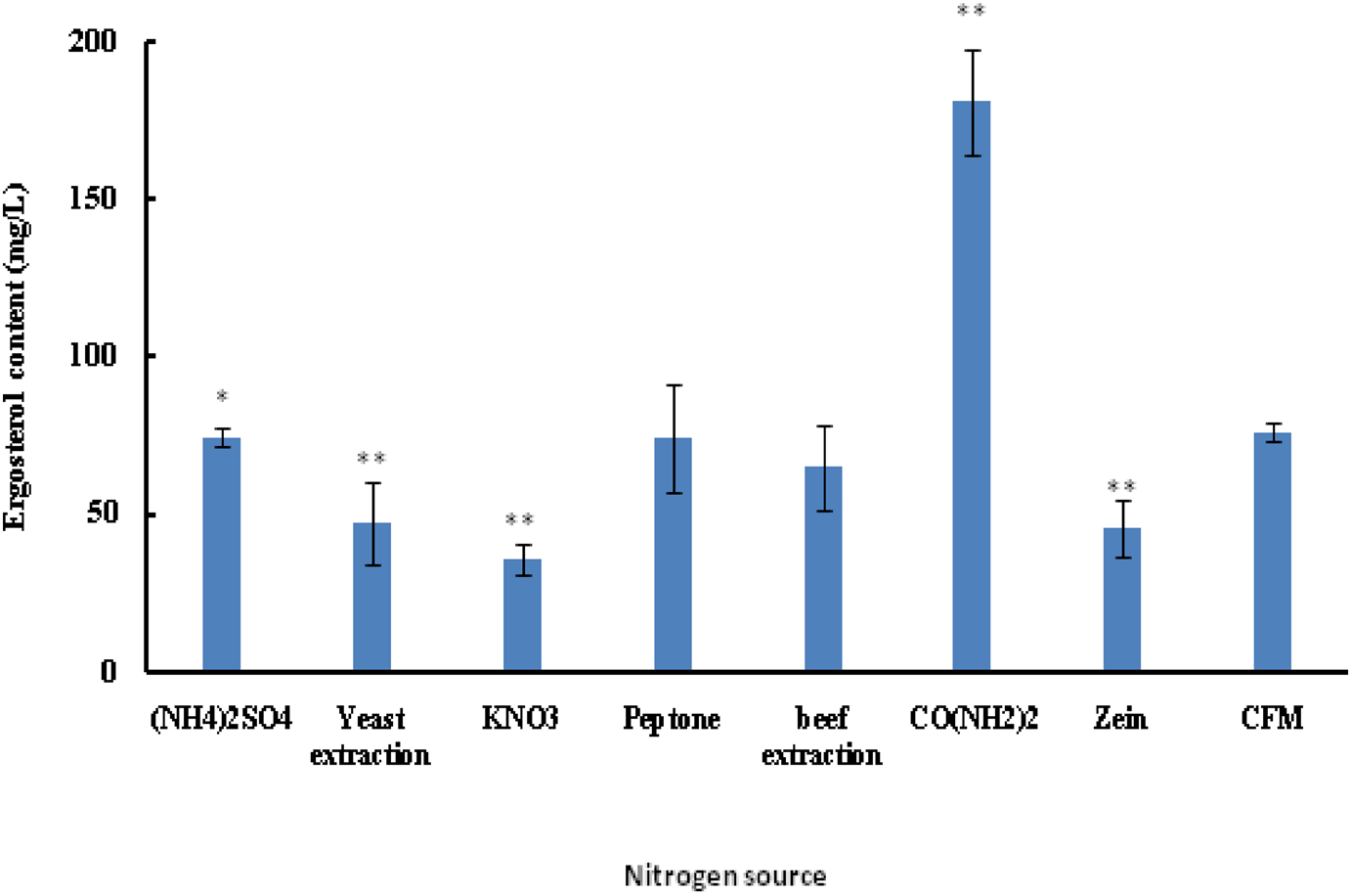
Effects of various nitrogen sources on the yield of extracellular ergosterol. CFM is the control fermentation medium.

In the fermentation process, nitrogen sources are not only an important nutrient for synthesis of nucleic acid, protein and vitamin in th**e** process of biomass growth, but also an important metabolic regulator for product synthesis. It is generally accepted that nitrogen sources are mainly used for the synthesis of biomass substances (amino acids, proteins, nucleic acids and so on) and nitrogen-containing metabolites. However, no information on regulatory effects of nitrogen source on metabolites is available. Therefore, we firstly analyzed the effects of various nitrogen sources on the yield of extracellular ergoserol synthesized by *C. cicadae.* Figure 1 shows that the ergosterol content obtained from the complete fermentation medium (CFM) and CFM + CO(NH_2_)_2_ were 76.01 mg/L and 180.7 mg/L, respectively. CO(NH_2_)_2_ could significantly increase the ergosterol yield.

Nitrogen metabolism is closely related to the biosynthesis of Secondary Metabolite. On the one hand, nitrogen sources can promote the synthesis of products. Caltrder and Niss reported that L-Cysteine and others amino acid used as nitrogen sources and could significantly enhance the synthesis of Cephalosporin^27^. On the other, nitrogen sources can inhibit the metabolite biosynthesis. Brakhage et al. reported that 100 mmol/L lysine supplied in the medium could reduce penicillin production by about 50%^28^. When other nitrogen sources used as a sole sources, they were obviously disadvantageous to the synthesis of ergosterol. Generally, the organic nitrogen sources are favourable for biomass growth and disadvantageous to metabolite synthesis. Our results was supplied the view. The reason led to the conclusion is the regulation of the amino acid and vitamins in the organic nitrogen sources.

### Thin layer chromatography (TLC) analysis

**Figure 2:**
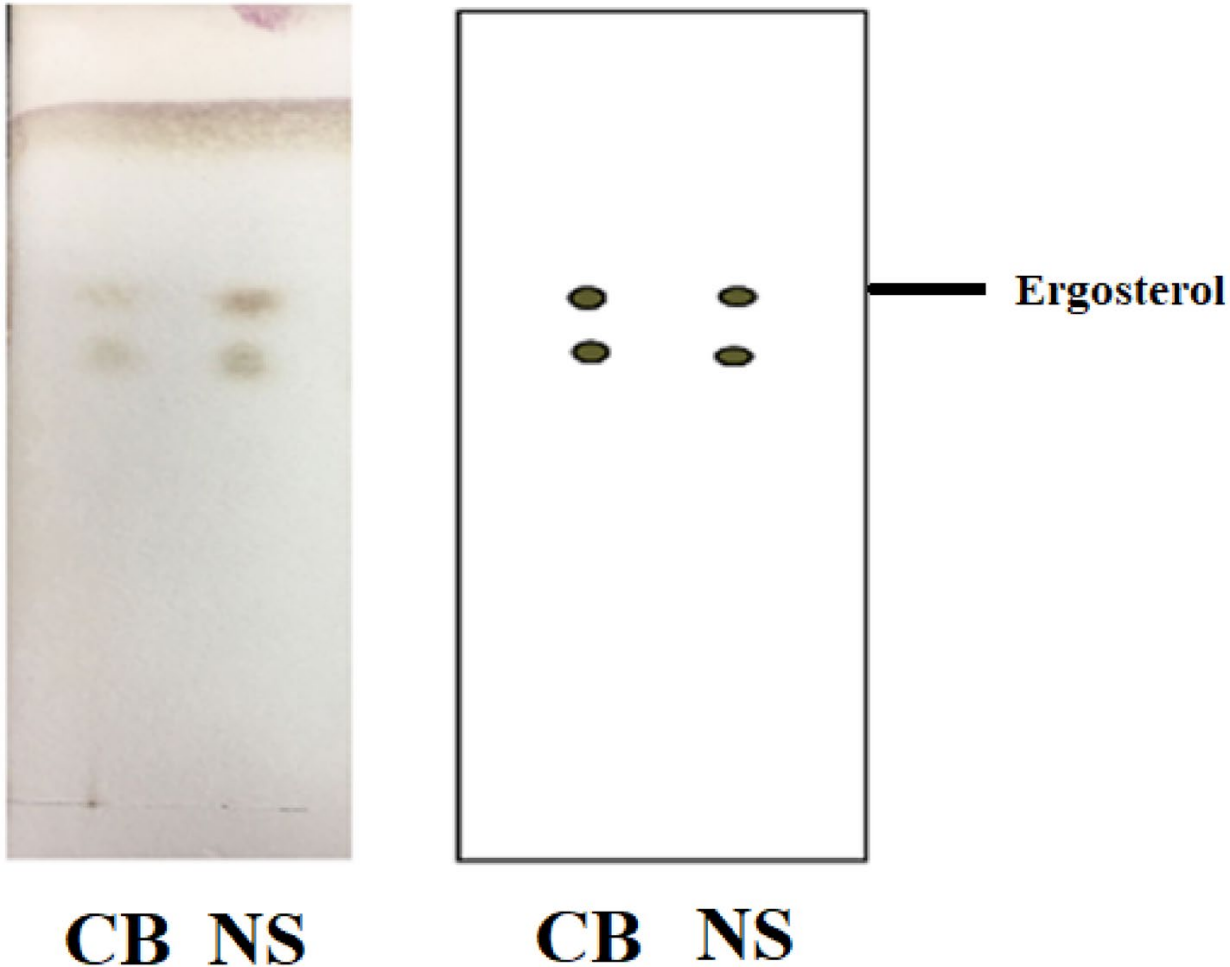
Silica gel chromatography of samples.

Many chromatographic approaches, including high performance liquid chromatography^29^, gas chromatography^30^ and TLC^31^, can be used for qualitative and quantitative detection of bioactive ingredients. Most methods require complex instrumentation which is expensive or in which data analysis is time-consuming. Among available methods, TLC is a simple and cost-efficient approach that is notable in ergosterol identification. Figure 2 exhibits that two spots appeared in the spectrum of control blank (CB) and CB + CO(NH_2_)_2_ (NS), and their corresponding R_f_ values were same, indicating that CB and NS contained the same ergosterol. Moreover, the colorization of NS was more obvious, revealing that the NS had more components compared with the CB.

### Transcriptome analysis of *C. cicadae* cultured in the CFM and CFM + CO(NH_2_)_2_ using RNA-seq

To investigate the key genes related to ergosterol synthesis by *C. cicadae* in response to CO(NH_2_)_2_, we performed transcriptome analysis of biomass cultured on the CB and NS for 94 h, respectively. Supplementary data 1: Table 1 summarizes the results of RNA-seq analysis, with three biological repeats from each sample. We obtained 5.94 ± 0.151 (× 10^7^) and 6.104 ± 0.398 (× 10^7^) raw reads from CB and NS based on 150 bases for each read, respectively. After removal of adapter-containing sequences and low-quality reads, we obtained 5.927 ± 0.150 (× 10^7^) and 6.084 ± 0.398 (× 10^7^) clean reads from CB and NS, respectively. The mass values Q20 and Q30 of raw reads of CB accounted for 98.057 ± 0.413% and 94.717 ± 0.890, respectively, and Q20 and Q30 of clean reads accounted for 98.177 ± 0.383% and 94.870 ± 0.854%, respectively. After urea response, Q20 and Q30 of raw reads of NS accounted for 97.847 ± 0.078% and 94.217 ± 0.179%, respectively, and the Q20 and Q30 of clean reads accounted for 97.973 ± 0.076% and 94.383 ± 0.168%, respectively. Q20 and Q30 showed that the proportion of obtained reads from samples that mapped to reference genomes was above 90%, meeting the requirement for subsequent analysis.

### The differentially expressed genes (DEGs) after addition of urea

To identify DEGs between the biomasses of CB and NS, we compared the two biomasses and screened for DEGs. In the experiment, the total number of assembled genes was 17,321, in which 17,208 and 17,203 genes were found in CB and NS, respectively. Supplementary data 2: Fig. 1 shows the length distribution of unigenes. In the 17,321 genes, 2,188 DEGs were identified. After addition of urea, 1,471 genes were down-regulated, and 717 genes were up-regulated (Supplementary data 3: Fig. 2).

To explore the function of unigenes identified in the *C. cicadae* transcriptome analysis, we performed a Gene Ontology (GO) enrichment assay. The GO enrichment pathway was mainly divided into three pathways: biological process, cell component and molecular function. Supplementary data 4: Fig. 3 shows the GO classification of differential enrichment analysis between CB and NS.

Supplementary data 4: Fig. 3 exhibits that there were 1,686 DEGs in this entry, and the number of DEGs annotated by GO was 1340, of which 675 DEGs belonged to the biological process analysis, 305 DEGs belonged to the cellular component, and 706 DEGs belonged to molecular function. These DEGs mainly acted on cell processes, metabolic process, biological regulation, membrane, combination, single biological process, transmembrane transport and catalytic activity. The down-regulated genes were mostly in the GO enrichment map.

### Functional classification of DEGs

The Kyoto Encyclopedia of Genes and Genomes (KEGG) is a database for analyzing the metabolic pathways and functions of gene products in cells. KEGG can be used to further study the complex biological behavior of genes. According to the annotation information of KEGG database, we could obtain the pathway annotation of unigenes. Supplementary data 5: Fig. 4 illustrates a KEGG enrichment map of CB and NS. After KEGG enrichment, the number of genes in the background gene set to the KEGG pathway was 2976, and the number of the genes in the specific gene set to the KEGG pathway was 630. The differential genes were mainly concentrated in metabolism. In the metabolic process, there were more differential genes in the following metabolic pathways. Global and overview maps had 199 differential genes, carbohydrate metabolism had 74 differential genes, and amino acid metabolism had 72 differential genes.

### DEGs related to ergosterol synthesis between CB and NS

**Figure 3:**
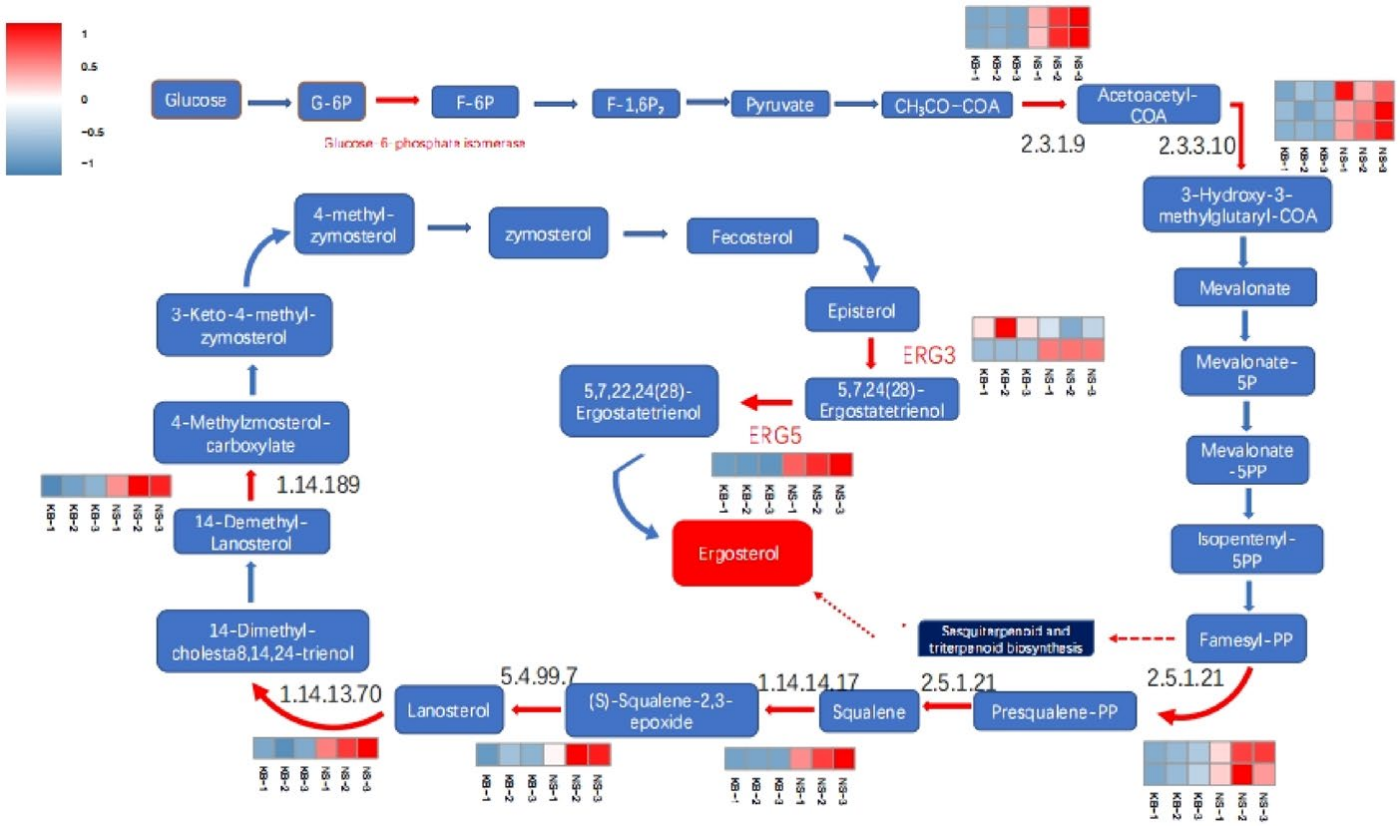
The pathway of ergosterol synthesized by *C. cicadae*.

The transcriptome is a collection of DEG of specific cells, tissues, and organs under the specific factors, such as the specific media component and culture condition. The DEGs of microorganism under the specific environments is therefore a matter of concern in transcriptome research of microorganism. In this study, we focus on the genes change in the ergosterol synthesis process by *C.cicadae* under the urea existing. The pathways of ergosterol synthesis could be divided into four stages. Figure 3 shows that there are significant changes in the expression of some key genes at each stages in the pathway. The first stage was the glycolysis of glucose. In this stage, the glucose was transformed into the CH_3_CO ~ SCOA. The second stage was farnesyl-PP synthesis. The third stage was the synthesis of (s)S-qualene-2,3-epoxide from the farnesyl-PP, and the fourth stage was the ergosterol synthesis.

#### The regulatory action of serine

In the first stage, glucose-6-phosphate isomerase (unigene 0010460) and D-3-phosphoglycerate dehydrogenase gene (unigene 0010219) were up-regulated, while enolase (unigene 0007238) and L-serine/L-threonine ammonialyase (unigene 0012663) were down-regulated. These changes of DEGs indicated the increase of serine synthesis. The increased free serine level in the cytoplasm can allosterically activate pyruvate kinase (PYK)^32^ and enhance the activity of pyruvate dehydrogenase (PDH)^33^. Therefore, we deduced that addition of urea in the medium increased PYK and PDH activity by elevating the plasma serine level. Serine was a signaling molecule that regulates tricarboxylic acid cycle. Consistent with previous findings^34,35^ that the increased level of free serine in the cytoplasm can down-regulate tricarboxylic acid (TCA) cycle and ATP synthesis, we observed that the citrate synthase gene (unigene 0003274), aconitase gene (unigene 0013230), isocitrate dehydrogenase gene (unigene 0007893) and isocitrate lyase gene (unigene0001163) were down-regulated. The changes of DEGs proved that TCA pathway and glyoxylate cycle were down-regulated, and CH_3_CO ~ SCOA synthesis was increased in the biomass of NS. Increased CH_3_CO ~ SCOA synthesis provided solid evidence supporting the increase of ergosterol synthesis.

**Figure 4:**
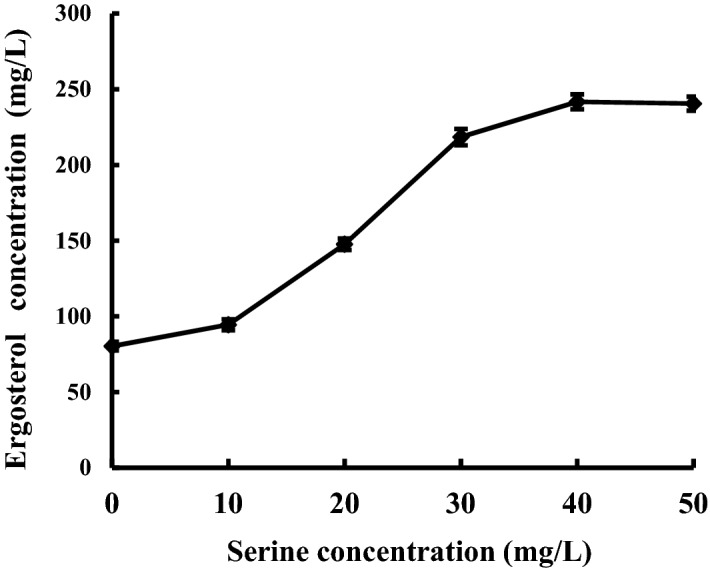
Effect of the serine concentration on the yield of extracellular ergosterol.

To verify the result, we carried out the shake flask fermentation added different 0, 10, 20, 30, 40 and 50 mg/L serine in the CFM. Figure 4 showed that when serine concentration increases from 0 to 40 mg/L, ergosterol concentration gradually increases from 80.3 mg/L to 241.7 mg/L. The result further proved that serine could increase the synthesis of ergosterol.

#### The regulatory action of SREBP

The second stage was the farnesyl-PP synthesized from the CH_3_CO ~ SCOA. The acetyl-CoA C-acetyltransferase (unigene 0007473) was the key enzyme of this stage. The enzyme catalyzes the condensation reaction of CH_3_CO ~ SCOA. Figure 3 shows that the acetyl-CoA C-acetyltransferase gene and hydroxymethylglutaryl-CoA synthase (unigene 00015804) were significantly up-regulated. The up-regulation of these two genes enhanced the synthesis of farnesyl-PP. In the third stage, the up-regulated genes included farnesyl-diphosphate farnesyltransferase gene (unigene 0007264), farnesyl-diphosphate farnesyltransferase gene (unigene 0007263) and squalene monooxygenase gene (unigene0013062). In the fourth stage, lanosterol synthase gene (unigene 0004466), steroid 14α-demethylase (unigene 0017047), methylsterol monooxygenase (unigene 0010744), δ-7-sterol 5-desaturase (unigene 0012727) and sterol 22-desaturase (unigene 0005363) were up-regulated. The up-regulation of these genes enhanced the yield of extracellular ergosterol.

Many fungi have been reported to have the SREBPs^24^. The striking trait of SREBP is that the sequence contains a unique basic helix-loop-helix (bHLH) DNA binding domain. The domain recognizes the SRE (sterol regulatory element) sequences of the genes that involved in ergosterol synthesis^36^. The vital role SREBPs play in the regulation of ergosterol biosynthesis and play important roles in other biological processes. For example, the *Cryptococcus neoformans* and *Aspergillus fumigatus* SREBPs have been reported to be related to hypoxia adaptation, fungicide resistance, and virulence^37,38^.

We found a gene similar to SREBPs. Supplementary data 6: Fig. 5 showed the the gene sequence. The gene sequence contained 4068 bp. Compared with the BLASTN library, the homology between the gene suquence and the sequence of Beauveria bassiana ARSEF 2860 helix-loop-helix DNA-binding demain was up to 78%. The gene similar to SREBPs might control these genes transcription levels including methylsteroid monooxygenase (ERG1), steroid 24-C-methyltransferase (ERG6), C-8 steroid isomerase (ERG2), δ-7-steroid 5-desaturase (ERG3), steroid 22-desaturase (ERG5) and δ-24(24(1)) steroid reductase (ERG4)^39^.

**Figure 5:**
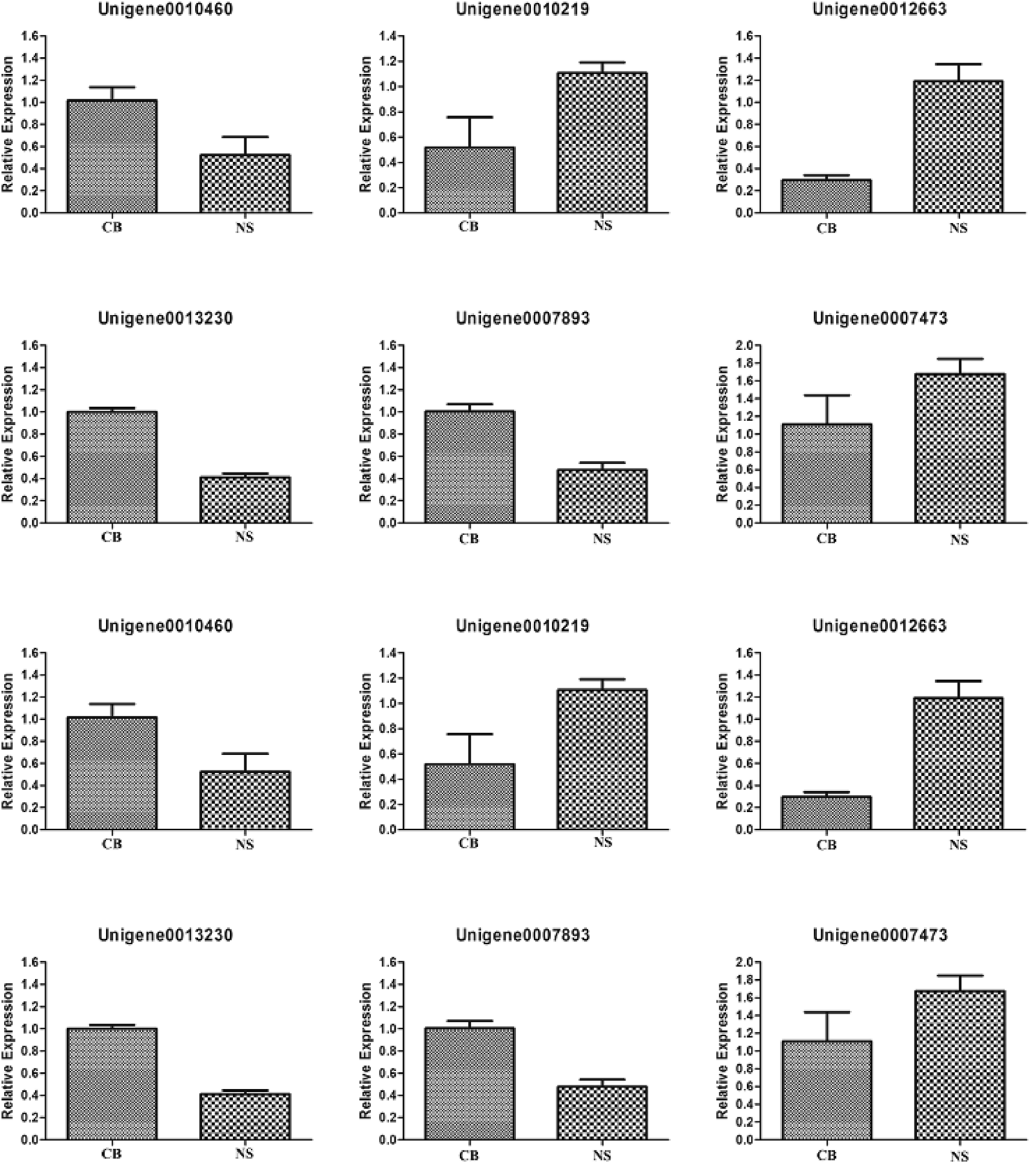
qRT-PCR verification diagram between *C. cicadae* myceliawith and without urea. Comparison of theexpression levels determined by qRT-PCR from three biological replicates. All data indicate mean ± standard error(SE). Unigene0010460, Glucose-6-phosphate isomerase;Unigene0010219, D-3-phosphoglycerate dehydrogenase; Unigene0012663, L-Serine/L-threonine ammonialyase; Unigene0013230, Aconitase; Unigene0007893, Isocitrate dehydrogenase; Unigene0007473, Acetyl-CoA C-acetyltransferase; Unigene0015804, Hydroxymethylglutaryl-CoA synthase; Unigene0013062, Squalene monooxygenase gene; Unigene0017047, Sterol 14α-demethylase; Unigene0010744, Methylsterol monooxygenase; Unigene0012727,δ-7-Sterol 5-desaturase; Unigene0005363, Sterol 22-desaturase.

To validate the transcriptome data, 12 DEGs were detected by qRT-PCR. The selected genes were mainly in Embden-Meyerhof pathway (Unigene0010460), Tricarboxylic acid cycle (Unigene0013230, Unigene0007893), serine synthesis (Unigene0010219, Unigene0012663), farnesyl PP synthesis (Unigene0007473, Unigene0015804), S-qualene-2,3-epoxide synthesis (Unigene0013062), and ergosterol synthesis (Unigene0017047, Unigene0010744, Unigene0012727, Unigene0005363). The results of qRT-PCR (Fig. 5) were consistent with the transcriptome data (Supplementary data 5: Fig. 4).

## Material and methods

### Strain materials

*C. cicadae* was gifted by the Key Laboratory of Food Science of Jiangnan University and maintained on potato dextrose agar (PDA) slant. The slants were incubated at 25 °C for 5 days and then stored at 4 °C.

### The preparation of biomass and filtrate of C. cicadae

The slant *C. cicadae* was inoculated into 250-mL Erlenmeyer flasks containing the seed culture solution. The seed medium was composed of (g/L) 20 wheat bran, 20 glucose, 10 corn flour and 4 fish meal peptone. These flasks were incubated on a rotary shaker at 25 °C and 150 rpm for 72 h. The seed liquid was aseptically transferred into 250-mL Erlenmeyer flasks containing 100 mL of fermentation medium at an inoculation ratio of 10%. The control fermentation medium (CFM) was composed of (g/L) 36 wheat bran, 46 xylose, 28 fructose, 25 glycerol, 1.7 ZnSO_4_ and 1.8 KH_2_PO_4_. After inoculation, these flasks were incubated on a rotary shaker at 25 °C and 150 rpm for 120 h. The tested nitrogen sources included urea, yeast extract, beef extract, zein, ammonium sulfate and potassium nitrate. The nitrogen sources were added at a concentration of 6.6 g/L. After incubation, the broth was centrifuged at 4,000 rpm for 10 min at 4 °C. The supernatant was collected to analyze the ergosterol content.

### The qualitative identification and content determination of ergosterol

Briefly, 25 mL supernatant was dried to constant weight at 70 °C. Subsequently, the residue was extracted by 25 mL 60% ethanol in a water bath at 75 °C for 4 h, followed by centrifugation at 12,000×*g* for 10 min at 4 °C. The supernatant was collected for qualitative identification and determination of ergosterol content.

The ergosterol was qualitatively identified according to TLC method. Briefly, 2 μL of sample solution was dropped on a TLC plate (2.5 × 10 cm). The plates were developed in vertical saturated chamber with mobile phase of chloroform–methanol-water-ammonia (25:10:5:0.8) to a distance of 8 cm. The developed plate was colorized with vanillin–H_2_SO_4_ solution (1% vanillin dissolved in 100 mL 10% H_2_SO_4_-ethanol), followed by incubation at 105 °C on a YOKO-XR plate heater (Wuhan YOKO technology Ltd., China). The spot of ergoesterol was recorded according to the indication of the standard ergosterol (Yunnan Xuli Biological Technical Co., Ltd).

The content of ergosterol in the broth was determined according the vanillin method. Briefly, 1 mL of sample solution was mixed with 0.2 mL 5% vanillin-glacial acetic acid solution and 0.8 mL of perchloric acid. The mixture was heated at 60 °C for 15 min, cooled to room temperature, and then reacted with 3 mL of glacial acetic acid. The absorbance was determined at a wavelength of 570 nm. Ergosterol was used as the standards.

### The qualitative transcriptome analysis

#### The biomass preparation

The biomasses cultured in the CFM and CFM + CO(NH_2_)_2_ for 94 h were collected by centrifugation at 10,000×*g* for 10 min. The biomass was washed with sterile water for four times and stored at − 80 °C until RNA extraction. The biomass cultured in the CFM was used as the control blank (CB), and the biomass cultured in the CFM + CO(NH_2_)_2_ was used as the sample of urea response (NS).

#### RNA isolation and sequencing

RNA isolation and sequencing were operated according to the reference^40^, with some modifications. Briefly, total RNA was extracted from CB and NS using Vazyme-innovation in enzyme technology reagent following the manufacturer’s instructions. Next, mRNA was purified from total RNA using Oligo (dT) beads, fragmented into short fragments using fragmentation buffer and reversely transcribed into first-strand cDNA with random primers. The second-strand cDNA was synthesized by DNA polymerase I, RNase H, dNTPs and buffer. Subsequently, the cDNA fragments were purified with QiaQuick PCR extraction kit, end repaired, poly (A) added, and ligated to Illumina sequencing adapters. The ligation products were size selected by agarose gel electrophoresis, amplified by PCR, and sequenced using Illumina HiSeqTM4000 by Gene Denovo Biotechnology Co. (Guangzhou, China).

#### Bioinformatics analysis

The obtained raw files (raw reads) were not clean, which contained jointed, repetitive, low-sampling reads that affected assembly and subsequent analysis. The clean reads of high quality must be obtained by removing the reads containing the adapter, the reads with a ratio of N greater than 10% and the low-quality reads (the number of bases with a mass value of Q ≤ 20 accounted for 40% of the entire read) from raw reads. These short clean reads were assembled from scratch into non-repetitive sequence unigenes using the short-read assembly software Trinity 2.1.1. The DEGs of CB and NS were analyzed by the digital gene expression (DGE) profiling. These unigenes were annotated to NCBI non-redundant protein (Nr) database (http://www.ncbi.nlm.nih.gov), the Swiss-Prot protein database (http://www.expasy.ch/sprot), the KEGG database (http://www.genome.jp/kegg), the COG/KOG database (http://www.ncbi.nlm.nih.gov/COG) and GO using BLASTx program (http://www.ncbi.nlm.nih.gov/BLAST/) with an E-value threshold of 0.00001. Functional annotations of proteins could then be obtained according to the best alignment results. GO annotation of unigenes was analyzed by Blast2GO software^41^. Functional classification of unigenes was performed using WEGO software^42^.

### Quantitative real time PCR (qRT-PCR) analysis

A set of DEGs identified in this research were selected for qRT-PCR verification for detection of transcriptional changes in details after salt and ethylene treatment. The selected sequence templates were listed in the Supplementary data 7. The Unigene0009896 was used as the endogenous control. The used primers were designed using Primer Premier 5.0 (Premier) and listed in Supplementary data 8.

### Ethical approval

This article does not contain any studies with human participants or animals performed by any of the authors.

## Conclusions

In the present study, we comprehensively investigated the mechanism underlying the urea-induced ergosterol synthesis by *C. cicadae*. The findings indicated that the expressions of many genes were changed after the *C. cicadae* was cultured in the presence of CO(NH_2_)_2_. The alteration of these genes played an important role in promoting the ergosterol synthesis. Serine was a signaling molecule that down-regulates tricarboxylic acid cycle and increase ergosterol synthesis by *C. cicadae.*

To the best of our knowledge, for the first time, we reported the regulatory mechanism of urea to ergosterol synthesis by *C. cicadae* based on Illumina RNA-Seq. Our finding provided a better understanding of the effect of urea on ergosterol synthesis.

## Supplementary Information

Supplementary Information.

## Abbreviations

CFM: Control fermentation medium
DEG: Different expressed genes
PDA: Potato dextrose agar
TLC: Thin layer chromatography
CB: Control blank
NS: Urea group
