# Supplementary material for: The transcriptome analysis on urea response mechanism in the process of ergosterol synthesis by Cordyceps cicadae
Source: Sci Rep. 2021 May 25;11:10927. doi: 10.1038/s41598-021-90377-2 (PMC8149685; doi:10.1038/s41598-021-90377-2)
Supplement: Supplementary file 1 — Supplementary Information. [file 41598_2021_90377_MOESM1_ESM.docx]

[**Supplementary data**](https://oup.silverchair-cdn.com/oup/backfile/Content_public/Journal/bioinformatics/35/23/10.1093_bioinformatics_btz299/1/btz299_supplementary_data.pdf?Expires=1579719599&Signature=CHIks1Rtr9Mctbmc67tW44aWhLMvRgeBvE3u-dkCM6Ykr4xxt6IWviQuYqPK4EG7N6nMo3YRqvy6ztsDH~GIxh50UEOvxITWcz~UpfVVh7Pj6bRF6NjSSdwDPes82FWoEMgRrGh3QjtMPPH5cWTf889N7y7hwG4EwM2vmLu98DKtkQP-Z5S5FObqyfnsbJdbIO~6hplV32bK9HdFukpGmeWFD6g-bXLYdER3U3J-hLqxWM~Iry-UmoG~s1Pmd9iEHEzrq1LOb4ahEf6QQpa3UC6hXX~UZiCb8KjfmBYBtqQiM9OMhn2o8WETf~n7N43ZFv6GqvfxqadDAk63oV7Igw__&Key-Pair-Id=APKAIE5G5CRDK6RD3PGA)

**The quantitative transcriptome analysis on urea response mechanism in the process of ergosterol synthesis by *Cordyceps cicadae***

Qihui Su^1^, Zhicai Zhang^2,3,*^, Xiaocui Liu^1^, Feng Wang^1,*^

^1^ School of Food Science and Biological Engineering, Jiangsu University, Zhenjiang, 212013, P. R. China

^2^ Institute of Agro-production Processing Engineering, Jiangsu University, Zhenjiang 212013, P. R. China

^3^ Zhenjiang Yemaikang Food Bio-Technology Co., Ltd., Zhenjiang 212013, P. R. China

Correspondence author: Zhicai Zhang, E-mail: [zhangzhicai@ujs.edu.cn](mailto:zhangzhicai@ujs.edu.cn); Feng Wang, E-mail: [fengwang@ujs.edu.cn](mailto:fengwang@ujs.edu.cn).

**Zhang et al. Supplementary data 1: Table 1:** Sequencing data for six libraries obtained by RNA sequencing

| Sample | Control | CO(NH_2_)_2_ |
| --- | --- | --- |
| Raw reads (×10^7^) | 5.945±0.151 | 6.104±0.398 |
| Clean reads (×10^7^) | 5.927±0.150 | 6.084±0.398 |
| GC (%) | 58.647±0.332 | 58.647±0.110 |
| Adapter (%) | 0.067±0.025 | 0.07±0.01 |
| Low quality | 0.243±0.075 | 0.26±0.017 |
| Raw bases (×10^9^) | 8.918±0.227 | 9.156±0.597 |
| Raw Q20 (%) | 98.057±0.413 | 97.847±0.0.078 |
| Raw Q 30 (%) | 94.717±0.890 | 94.217±0.179 |
| Clean bases (×10^9^) | 8.867±0.224 | 9.102±0.592 |
| Clean Q20 (%) | 98.177±0.383 | 97.973±0.076 |
| Clean Q30 (%) | 94.870±0.854 | 94.383±0.168 |

**Zhang et al. Supplementary data 2: Figure 1:** Length distribution of *C. cicadae* Miquel unigenes


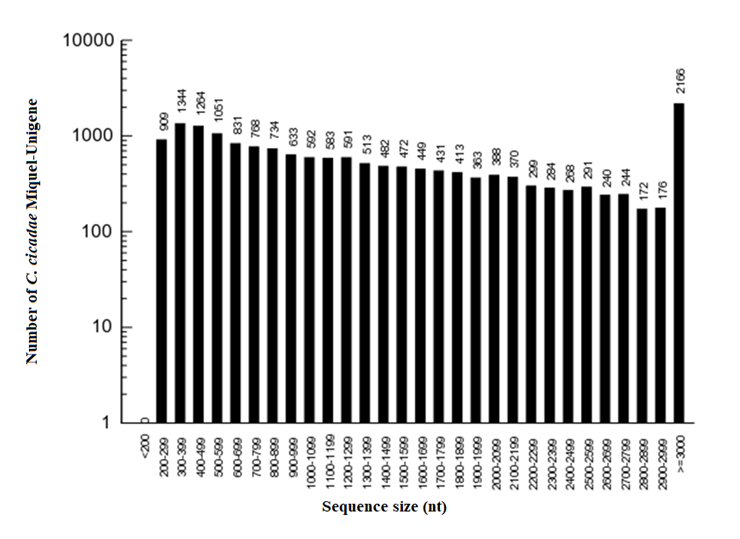


**Zhang et al. Supplementary data 3: Figure 2:** Different gene expressions in response to urea

**
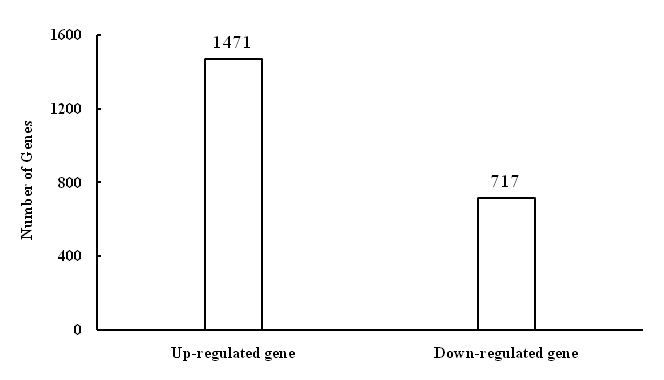
**

**Zhang et al. Supplementary data 4: Figure 3:** Annotation classification of unigenes by GO. ■: Up-regulated gene; ■: Down-regulated gene.

**
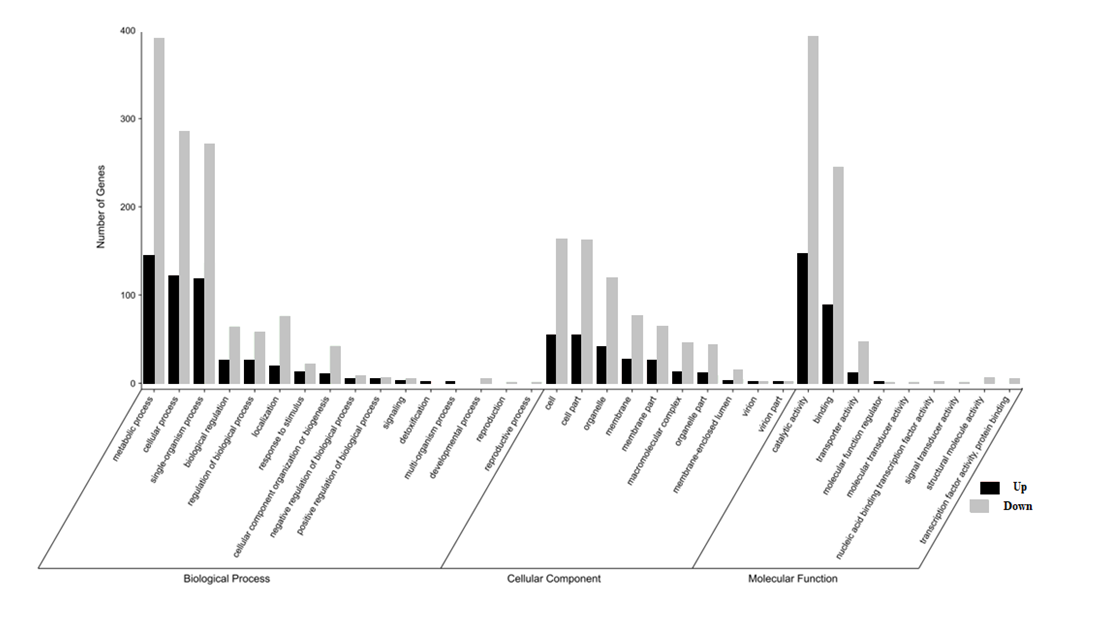
**

**Zhang et al. Supplementary data 5: Figure 4:** Differential enrichment analysis of KEGG enrichment of KB and NS


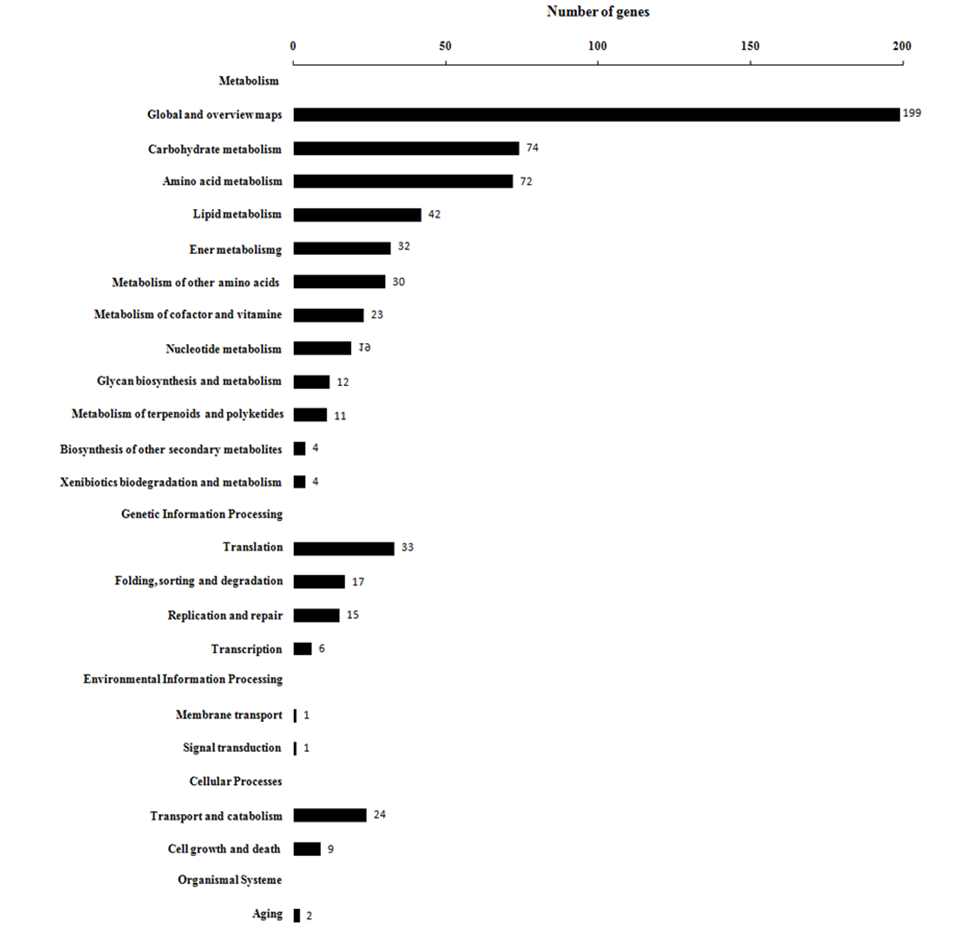


**Zhang et al. Supplementary data 6: Figure 5:** Gene sequence and comparison results of Unigene0010263

**Gene sequence**

GGGGGGGGGGGGAATCTACCAATGCTATCTGTACATGGCATTGAATAACCTAGTTACAAAAAACCATATCCAGAGAAATGAGGAAAGGGAGGAAAATGCATGAAAATAACGCCGTCATCCGAGAACATGGAACATGGAGCCCGCAACAGATTTTTTTTTTGAAACATCTTGTCAAGAGACCAAAAGAAAGAAAGAAAGAAAGCAAAAGAAATTCGTCCATCCGTTTTTCCATCTCCCGTCTCGATTCGTCCTTGTTTTATCCCACCATATCGTTGTTATACAAAAGCCAGAAGAAAATATGTCACGGTGAGTGAGGCAAAGCCCAGACCCCGGAAAAAATGCCGATAAGATGCGAAACCCCGTGTTCATTTGTGTCGTGTAAGCAAAAATACGAACAAACGTTAAAGGGAAGGAGCGTAGAGAAGAAAAAAAGATGACAGGTCAGAGCGGGGAGGGGGAATCATTTCGTGTTGGCTAGCGGGAGTCATCGTCGCTGACATCGCTAAGCGTTCCATAGCCCGTGTCGACATCCATGCCGACTAGGCTCTTCGTAATGGCTAGACAACGCTCAACAAGCTTGGCGCGGGTGTCGGCGTCGATGCCAGCATTGGAGCCGCAAGGGCCTCCGATCCACACGCGGAGTTTGGCCGCGAGCTTCTCCAGTGAGGCATCCACAGCATCGTCTTCGCCGCGGTGCTGGGCCATGACTCGCTCGAGTAGTGTCATGACGGCCGTGAAGCCAAGAAGGGTCATGTTTGAAGCCATGGCGGGACGCAGCGAGGCCTCAATGACACGGAGACTCTTGCATGGAGGCACTGCGGCGTCCCGGCCGCGCGAGAGCTGGCCGAGGGCCACGGCGCTTCGCAGGGTTAGTAGCAAGTCGGGGTTTGGTGCGTGGATGCTCGAGCCGATGACGACCTTGGACTCTTGTTTTGGCGTCTCCAGGCGCTCAGTTCCTAGCAGAGTCAGCGACGAGGCGATACTGGCCCCGCGAGACTCGTCGACGAGGGTGGCGCAGGCCAGAGAGGCGCGTAGCTTGGCGATGGAGCCTGGCGGGGCGACGCTGAGGGCCGTGTTGAGGTCCTCTTTGTTGACCTTTTTTGTGTCGAGGGCATCGGTGAGAACATCGTGCAGGAGGCAGGTAGAGCACCAAGCGGCAGCCGCATCCAGGGGGGTGCCGACGTAAATATCGTATACGACAGAGTCCATGCCGTCAATTTTAGGCATCGCCCCTTCGGTAGCGTCTTTGTGAAAGGCGAGGTTGTATGCGCGGTGCACAACAGCATCAGTGAGGACATCGTCACAATCACGCTCCACTAGGGCGACGAGGTGCTCGGGAAGCTCATTCTCGTGTGGCTTCGTAGGGTCCGTTCGGAGCTGAATGAGAAGCTGATTGAGCTGACGAGCGCTGTTCCATCTAGACCGAGCAATTTTGGCGGCAATCAAGTTGGCAGGTACAAACCTCGTCTGACCGAGCCTCCATAGCAGCACGCGAATATGCAGAGCCTTGAGCATGAGTCGCACTGGCGTGTCGGGAAGTGTGTCCGAAGCCATGAGAGTCAACAGTAGGCGGCGGGTGCAGACCTCAGAATCACCTCCAGCAAGTTGTGAGTCGAGTGCAATAGACCAAGCCTTGACGCGGGCTGCTTCCTCTTCGTGAGTTGTGCCGGTTAGCATTTGGTAGCCATTCCAGCCAATGGCGTTGCGTAAACTAAGCTTGGCCATCTTGAGCAGCAGGGCAGCAACCTCGAGGAAGAAGTTGTGGCGAGGCACCCAGACGGTCTGAACGGCTGTGGCCCAGGCCTGTCGTCGAACGTGGATCGGGGCTGCCATGGAGGGAGCGGCACGCAGTTGATCAGTACCCTGCTTCTTGGGGTTGCTGTCAGAAGATTGGAAGAGCGAAGGCACAAAGATCCAGAGGAAGACGCCGAGGAACAGGAGGGATTGAATGGAGGTGTGAATGTGGTATCCGTGGTAGTTGAAATCGAGGCCCTTGGCAGCAGTACGGAGCAGCTGCACGGGAATGGCAGCTAGACCGCGACCTTCTGGGGTTTCGTTGCTGACCTCGTCCTCGCGGAAAGCTTCGATGATCATGAGACCGGCGAGGGAACCAACCATGAGCTTGCCAAAGTAGGGGCTGGCATTGGACCAAGCACCTTGTTGCTGCTGGACTTGCTGCTGCTGGATCTGCTGTTGGCGAACAAGGGCGGGGTTGGCTCCCTGGAAGGCTTGCTGTTGCGGTACTGGATACGGCTGTCCAGCAGCCATTTGGGCGGCGAGGATGCGCCTCATGTCGTCGGGCACCTGGATCATGCCAGCAGGCGTCTCGCCGTTTTCTTGTTGGGGAATCGGGCTCTGCATGAAATTTGACTGGCCTTCCTGCGGGAAGGGCATTTGCGGAGGTGGTTGATGGGAGCTGATGGAGCCATTCATGGCGCCAGCCATGAAGAGCTTCTCAAAGGCAGCTATCCTGGCCTGCATGGCGTTGTTCTCTTCCAACAGCCTAGTGTTTCGCTTTTCGAGATGGCGGATATATTCGGTAGCCTTGCTGAGGACCTGAAAGATTACGTGATCAGCCTGGATCTATAGACGGTGGGCTTGCAACATGTCATACGGTAGCCTTATTTAATTTGTGTGCGGGGGTGAGGCCGTGAAGTTCTTCACGATCCTGGGTGGTATCTTCGCCGCGGGCGCTCTTGGACATGATGCGTAGGCTAGGAACGCTGTCTCGTAGGGCGGCAATCTTGTCATTGATGTTTGTTCTGTAGCGCTTCTCAATCATGTTGTGTGCAGTCTTCTTGACAGGCTTGTCCTCATCTTCGGCATCATCCTCTTCTTCGGGCTCTGAAGATACCTTGCGTTTTCTCGACTCGTGGTGTCTCGCTTCGGAGGCGTCTTCGGGGGAGGAGATGATGCCGGTCTTGGTTTCGCTCTTGGAGGAATCAGGGCTGGACATGTGAAGGATTTGGCGGGGTAGACCTCCAGGCATGGCGATGTTTCGAAGCTTCTCCTCGACGGCCGGGGCCAGCGAGGTGGAAAAACGAGGTGGTTGGGCGACGGGCTGGCGGGAGCTGGCGTTTGAAGCCCTGCCGTCTTCGCTAGGTGCGGTGGGTGTGAAGCCGTTGGGATCTGAGCTGTTGGAGAAGAGGTTGATGGGGTTAACGCCAGCGGAGAGTGCCGACTGTGAGGAGGCAGATGAGGTCTGTGGTTGGGATTCGAAGCCTTGGAAGGACCAGGGCTGGGGAGTGAAGTCGGGTTGGGAAGCGGAGTTGCTGGGGGTGGTCAGGTTATAGGATTCCAGGCTGGAGGTGCCTTGAAAGTCCCAGACGCCGTTGGAGTCGAAGAGGGACTTGGTGATGTCGGAGGCGATGAGGGGTTGTTCGGGGGTGGCGGCGGTGGTGGTGAAGACGTCGTTGAAGGACATAGGACCATCTTCGAGAGCCAGCTGGCTGGCATTTTCGTCTGTGGACATGGCTACGTGTGGCCGTATGTGTGTGAGAGCAAACAAGACCCCTTTGGGTTGATGGCCGTGAGTTGGCGGTGCGGCGGAGCTGGGCTGCTGGACTGCTGGACTGCTGGGCTGCTGGGCTGCTGGGTCGCTGGGTCGCTGGGTCGCTGTGTTGCTGGCACGAGCAAAAAAGTCACTCGATTGTGCCCCCAGACGGGCAGGGATTGATTATAGCAGGACTCCGGGCCGGGCAGGCGTGCGGTCGAGAAGATGCGAGCCCAGGCGAGAAGGGTGGCGGCGAGCGGGCAAGAGCGGGCGAGAAGCGGAACCGGCGTGGTCGTCGATCAGGATAGGGCCGGCCGCTTTCTCCCGGGTAGAGGGCTGCAAGGCGTGCGGAATGGGCGCAGCAGCTGGGCGGTAGCAAGCAATAGCAGATGACTGCTTGATTGATTGACTGGGTTTTGCAGATGATGTCGGCAGTTGGGGGATGGCGTCGTCGGCGGGATGCGGCGAAGAGGATGT*GTGTCGGACG*ACTTTGGCACTAATGGGGTGATTTGGGTGACGGAATAGATGAAGAGGGACAAGGTAACTAGCTGGTATCGGGGTGATATGCATGAAGAAAGTTTTTTTTTTTTTTTTT

**The results of the comparison
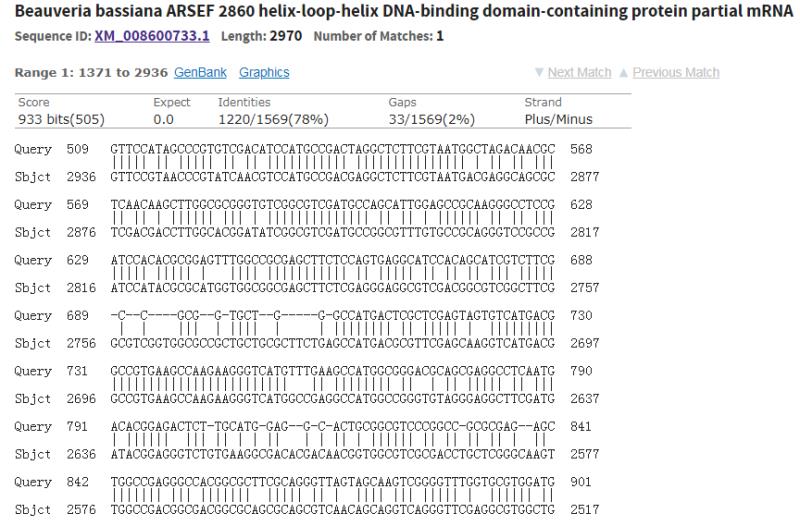
**

**
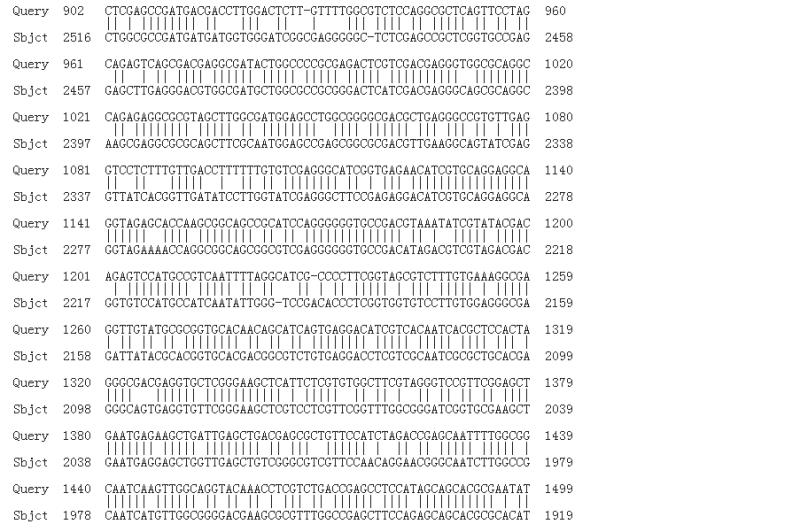

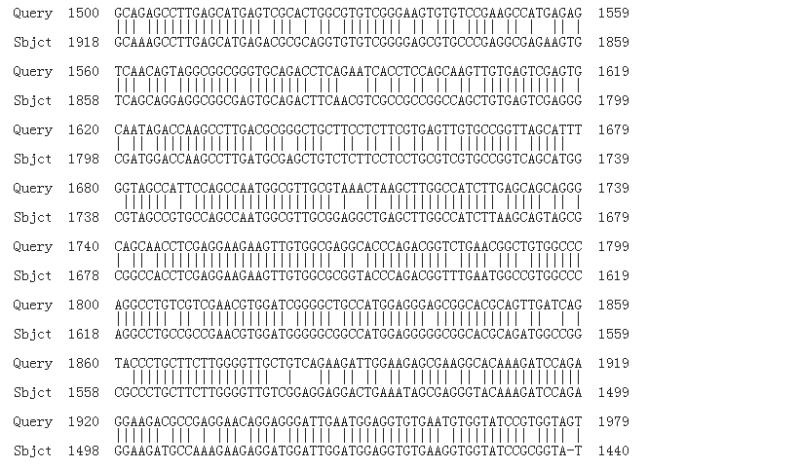

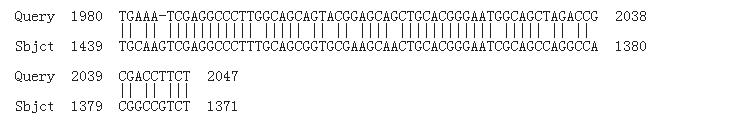
**

**Su et al. Supplementary data 7: Table 2: Temple used**

| Gene | Name | Metabolism pathway |
| --- | --- | --- |
| Unigene0010460 | Glucose-6-phosphate isomerase | Embden-Meyerhof pathway |
| Unigene0010219 | D-3-phosphoglycerate dehydrogenase gene | Serine synthesis |
| Unigene0012663 | L-Serine/L-threonine ammonialyase | Serine synthesis |
| Unigene0013230 | Aconitase gene | Tricarboxylic acid cycle |
| Unigene0007893 | Isocitrate dehydrogenase gene | Tricarboxylic acid cycle |
| Unigene0007473 | Acetyl-CoA C-acetyltransferase gene | Farnesyl PP synthesis |
| Unigene0015804 | Hydroxymethylglutaryl-CoA synthase | Farnesyl PP synthesis |
| Unigene0013062 | Squalene monooxygenase gene | S-qualene-2,3-epoxide synthesis |
| Unigene0017047 | Sterol 14α-demethylase | Ergosterol synthesis |
| Unigene0010744 | Methylsterol monooxygenase | Ergosterol synthesis |
| Unigene0012727 | δ-7-Sterol 5-desaturase | Ergosterol synthesis |
| Unigene0005363 | Sterol 22-desaturase | Ergosterol synthesis |

**Su et al. Supplementary data 8: Table 3: Primer used**

| Gene | | 5'→3' | T_m_ (℃) | Product (bp) |
| --- | --- | --- | --- | --- |
| Unigene0010460 | F | AGAAATGAAGCCGCAGACGA | 60.8 | 428 |
|  | R | GGCGAGTTGGGTAGGAATGT | 58.9 |  |
| Unigene0010219 | F | GGGCGAGTTGATGACGATGA | 61.3 | 229 |
|  | R | GCTGACCCCGCACTTTGA | 59.8 |  |
| Unigene0012663 | F | GCCGCAGACAATCAGGACA | 59.8 | 215 |
|  | R | GGGCTCACTATCCCAACACA | 57.9 |  |
| Unigene0013230 | F | GTCGGCAAAGGTGAGAGGA | 58 | 168 |
|  | R | AAGTGGGTCGTTGTTGGTGA | 58.3 |  |
| Unigene0007893 | F | CTATTGCCTGGGAGCCTGTC | 59.6 | 279 |
|  | R | AGTATTCGCCCTCGGTGTTC | 59.1 |  |
| Unigene0007473 | F | CCTCCTCGGGCTTGATTCC | 61.6 | 286 |
|  | R | ACAAACACACTCCTCGCCTC | 57.2 |  |
| Unigene0015804 | F | ATGTCTTCACGGTCATCGCA | 59.3 | 170 |
|  | R | AAAATGGCTGCTCGTCCTCA | 60.6 |  |
| Unigene0013062 | F | GGAGAGAGAAGACAGCACGG | 57 | 224 |
|  | R | GCCTACCACTTCTTCTCCGTC | 58.1 |  |
| Unigene0017047 | F | GCCGAAGAGGACAAAGGTGA | 59.9 | 138 |
|  | R | TCAAAATCAGAGCCGCCAGT | 60.6 |  |
| Unigene0010744 | F | CCAAGCAGGACAATCGGAGA | 60.7 | 278 |
|  | R | TTCTTCCACCCTATCGCCAC | 59.8 |  |
| Unigene0012727 | F | CGGGCAGATGAAGACGAAGA | 60.9 | 116 |
|  | R | TCCACAAGCCTCACCACAAG | 58.9 |  |
| Unigene0005363 | F | ATCAAGAACATCGGGTCGCT | 59.4 | 456 |
|  | R | GTCTCCTGTCGCACTTTCGT | 57.4 |  |
| Unigene0009896 | F | CGCACCCTCTCTGACTACAA | 55.9 | 270 |
|  | R | GACGCCAACATCCACACAAC | 59 |  |
